# Monthly Summary

**Published:** 1877-06

**Authors:** 


### MONTHLY SUMMARY.

---

*The Local Action of Chloral Hydrate.*—In an article in *L'Indipendente*, No. 6, 1876, Dr. Guido Tizzoni, of Pisa, observes that the first effect of the application of solution of chloral hydrate to parts deprived of epidermis and epithelium is a transient irritation, varying in degree with the strength of the solution. This is followed by an anæsthetic action, in regard to which the observations of Drs. Coignard, Horand and Puech, have been confirmed by the author.

The irritant action favours the development of cellulo-vascucar granulations, and accelerates the cicatrisation of solutions of continuity. The chloral acts also as an astringent, and as an antiphlogistic. The irritant action, which is generally of brief duration, having ceased, there is in a few hours an arrest of the inflammatory processes, including suppuration: this appears due to the retardation of the circulation and the constriction of the capillaries, as well as to minute coagula and embola, which are found in the vessels and cut off or diminish the local supply of blood— a fact which has been established by the experiments of the author and of Dr. Fogliata.

The most important properties of chloral in surgery are those which it possesses as a disinfectant, antiseptic, antiputrescent, and antifermentative remedy. The author has used it for the preservation of flesh, milk, urine, etc.; no change being perceived at the end of two months. He recommends chloral in ordinary surgical practice, as a preservative against local and general infection, and as a restrainer of suppuration.

He describes the experiments and clinical observations which he has made with regard to the action of chloral, especially as an antiputrescent. The ultimate results were always the same, differences depending only on the amount of chloral used. The solution which he employs is one of ten parts of chloral in fifteen of water.

He has treated, both in hospital and in private practice, many cases of acute and chronic urethritis with injection of chloral hydrate, in the manner recommended by Parona, and has found the result to be rapid cessation of the discharge, and cure of the disease without complications.—*Lond. Med. Record.*

---

*Carbolized Camphor and its Therapeutic Applications*—Dr Soulez, of Romorantin, has a paper on this subject in the *Bulletin Generale de Therapeutique*, Aug 30. Desiring to combine the valuable properties of carbolic acid and camphor and founding his proceedings on the property possessed by certain acids of liquefying camphor, Dr. Soulez poured 2 grammes (30 grains) of carbolic acid solution (in the proportion of nine of carbolic acid to one of alcohol) on 12 grammes (180 grains) of camphor in powder, in successive portions, taking care to keep the mixture agitated. This produced a liquid of a syrupy consistence. Carbolic acid dissolves a larger quantity of camphor than that just mentioned, but in practice Dr. Soulez contented himself with a solution of 2.50 grammes (37½ grains) of powder for 1 gramme (15 grains) of acid. The product thus obtained is an oleaginous liquid with the combined odour of camphor and carbolic acid, which boils at a slightly elevated temperature without decomposing, does not mix with either water or glycerine, is decomposed by concentrated alcohol with a deposit after some hours of crystals of camphor, is miscible in all proportions with olive and olive oils, if poured in a boiling state into cold water immediately solidifies into a mass, and produces a red colour on the addition of nitric acid.

For dressings, Dr. Soulez uses mixtures of a twentieth part of carbolized camphor, either with olive oil or with infusion of saponaria (100 grammes of saponaria leaves with 1000 grammes of water) which makes an emulsion. A square of wadding soaked in the mixture of carbolized camphor and olive oil is placed in contact with the wound, and over this six other squares soaked in the emulsion of carbolized camphor and infusion of saponaria. To prevent evaporation, these are then covered with a thin layer of gutta-percha tissue, and the whole is secured by a piece of dry wadding as a bandage. It should be added that the whole wound, whatever be its nature, is washed before being dressed, with the emulsion. Thus applied, says Dr. Soulez, this dressing realizes all the favourable conditions inherent in Guerin's and Lister's method without its inconveniences. Usually the dressing is renewed every six days, sometimes being left in its place ten days, and up to the present time not the least irritation of the sk n or of the wound has been observed, which could be attributed to contact with the carbolized camphor.

Dr. Soulez then proceeds to narrate some cases in which this preparation was successfully employed, and concludes by drawing attention to the following as the advantages derived from the employment of the carbolized camphor: 1. Diminution in reaction after great operations; 2. Cessation or lessening of pain; 3. Less abundance of suppuration.—*London Med Record.*

---

*Rubber—Celluloid.*—I wish to say a very few words in regard to the above, at the beginning of this new year.

After four years trial with celluloid, myself and many of my co-laborers find ourselves disappointed in its use. We make more failures in fits than we did with rubber. We have to grind a great deal, after we think we have a perfect articulation; and we find that the color fades. We find that many of the plates warp so as to be useless. We feel that we would be glad to go back to the use of rubber if it was not for *fighting the patent.*

We even think that we would take out a *license* and cease the warfare if Mr. Bacon would sell us licenses without asking us to swear that we had not used celluloid.

During the last four months several young dentists have asked my advice about taking a rubber license. I advise them to do it, both for their peace of mind, and the benefit of their patients. Really, as for *myself*, I have ceased to have any *money* interest in artificial teeth.—*Editor Missouri Dental Jour.*

---

*The Preliminary Education of Medical Students*—There is a slight inclination on the part of a very few medical schools in the United States not to accept as a student a very grossly ignorant young man. The school at Portland, for instance, requires an applicant to know how to read and write English—not correctly, of course, but in some degree correct. In most institutions, however, it is entirely unnecessary that a matriculant should be able to do more than sign his name. He can enter, and it is possiple he can get through with no more than this.

Medical societies, therefore, would do wisely to take the initiative in this matter. It is gratifying to see that one in this State has done so, that of Alleghany county. The following is an extract from its by-laws:

" At the stated meeting in January, annually, the President shall appoint the following committee:

" A committee of three, to be called the Committee of Medical Examiners, whose duty it shall be to examine all applicants for admission as students under the tuition of the members of the Society. Said committee shall withhold their certificate from any applicant unless he be of good moral character, possess a good English education, and a sufficient knowledge of Greek and Latin to enable him to pursue his studies with advantage.

" And no member of this society shall receive any person as a student of medicine unless he presents a favorable certificate from this committee."

In one of our British exchanges the medical faculty of Glasgow University show the necessity of preliminary examinations, by quoting answers lately submitted by applicants. The best of these was that of a gentleman who understood the " Letters of Junius" to be letters written in the month of June.

But we need not cross the water to find such instances. Let it, however, be generally understood that medical societies are going to take such action as that above set forth and these examples will become rare.—*Med. and Surg. Rep.*

---

*Blue Glass.*—Nothing of a therapeutical character has made such an excitement among the non-professional public for years as General Pleasanton's Blue Glass Cure is creating just now. It is *the* topic in social circles, and a house with a southern window is not considered to be furnished until it has a foot square of blue glass suspended in it. The experiments are said to be most popular among those ladies whose adipose tissue is rather scant in comparison with their osseous development, and great things are in anticipation. Now is the chance for some of our pharmaceutical friends to make hay while blue sunshine is in fashion.—*New Remedies.*

---

*Advice to Students.*—A distinguished English Professor gives the following advice to students on the subject of examination: "It is unnecessary to advise you to avoid studying merely by way of preparation for examination. Work diligently, study thoughtfully and deliberately, above all, be thorough, otherwise your knowledge will be unaccompanied by that enlightenment of the understanding, that mental training, discipline and general elevation of the intellect which constitute in a word education. When you are thus educated, you will with ease and pleasure pass any examination in the knowledge you have thus acquired. All authorities on education, whether statesmen, teachers or examiners, regard 'Examination,' even by the most highly skilled Board, with ample time at its disposal and a wide area from which to select questions, as but a partial test of knowledge, and an extremely imperfect test of education."

The cramming process may do for a Freshman who has the whole course of study before him, and even though conditioned, by dint of hard study he may make up the deficiency; but an examination which has in view the entrance on a professional life, and such as involves great responsibility, can not be passed so lightly.—*Med Eclectic*

---

*Stimulants Used by the Race*—It is estimated that coffee, both beans and leaves, are drunk by sixty millions of the human frmily. Tea of all kinds is used by five hundred millions, and opium by four hundred millions; alcohoi, in its various forms, by five hundred millions of the human race. Tobacco is used by seven or eight hundred millions. These starting facts indicate a large proportion of the race using some substance that are either stimulants or narcotics. The work of the physiologist, in the future, will be to determine the true place in nature of these substances, and indicate where their use ends, and abuse begins.—*Quar. Jour. of Inebriety.*

---

*An Iron Calculus.*—A man in Paris recently passed a urethral calculus the size of a hazel-nut. Its passage was attended by severe nephritic colics. M. Cazeneuve found on examination, that it was entirely composed of almost pure peroxide of iron.—*Med. Record.*

